# Zero-Valent Iron Nanocatalysts via Polymers or Metal Hydroxide Passivation: Implications for Advanced Oxidation Processes

**DOI:** 10.1021/acsanm.5c04286

**Published:** 2025-11-29

**Authors:** Carlos Díaz-Ufano, Nahuel Nuñez, Alvaro Gallo-Cordova, Elin L. Winkler, María del Puerto Morales, Sabino Veintemillas-Verdaguer

**Affiliations:** † Departamento de Nanociencia y Nanotecnología, Instituto de Ciencia de Materiales de Madrid, ICMM/CSIC, C/Sor Juana Inés de la Cruz 3, 28049 Madrid, Spain; ‡ Escuela de Doctorado UAM, Centro de Estudios de Posgrado, Universidad Autónoma de Madrid. C/Francisco Tomás y Valiente, 28049 Madrid, Spain; § Gerencia de Física, 549773 Laboratorio de Resonancias Magnéticas, Centro Atómico Bariloche, Av. Bustillo 9500, 8400 San Carlos de Bariloche, Río Negro, Argentina; ∥ Instituto de Nanociencia y Nanotecnología, CNEA/CONICET, Nodo Bariloche, Av. Bustillo 9500, 8400 San Carlos de Bariloche, Río Negro, Argentina; ⊥ 42670 Instituto Balseiro, CNEA-UNCuyo, Av. Bustillo 9500, 8400 San Carlos de Bariloche, Río Negro, Argentina

**Keywords:** zero-valent iron, coating, stability, reactive oxygen species, Fenton-like catalysis

## Abstract

Zero-valent iron (ZVI) nanocatalysts are promising materials for environmental remediation (e.g., wastewater treatment) via advanced oxidation processes (AOPs) due to their ability to generate reactive oxygen species (ROS). However, their high reactivity often leads to rapid surface oxidation and a limited stability. This study evaluates the effect of different surface coatings on the stability and ROS production of ZVI nanocatalysts. The coatings tested include organic molecules such as mannitol and polyvinylpyrrolidone (PVP), as well as combinations with thin layers of metal hydroxides (M­(OH)_2_, where M = Ni or Mn). Hydroxyl radical (^•^OH) generation was quantified using electron paramagnetic resonance (EPR) spectroscopy over a period of 0 to 7 days in acetate buffer (pH 5). Results show that incomplete PVP coverage leads to a 90% decrease in ROS production within 24 h, while full coverage preserves catalytic activity for at least 1 day. Among inorganic coatings, Mn­(OH)_2_ maintained stable ROS generation for 7 days, whereas Ni­(OH)_2_ showed significant degradation after 24 h. These findings highlight the importance of coating selection and surface passivation to enhance ZVI stability and extend its catalytic performance in AOP applications, providing design guidelines for nanocatalysts in Fenton-like processes for water purification and environmental remediation.

## Graphical abstract

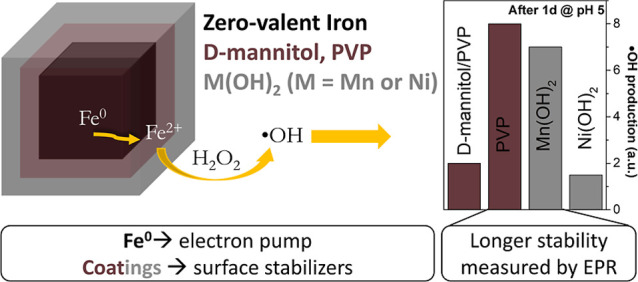

## 1 Introduction

Nanotechnology plays a crucial role in environmental remediation due to the unique physical and chemical properties of nanomaterials, such as a high surface area-to-volume ratio, which increase their reactivity and capacity for pollutant adsorption and degradation. This is the case for zero-valent iron nanoparticles, that can effectively break down organic pollutants, immobilize heavy metals in the soil, and remove contaminants from groundwater.^1–3^ This versatility makes them valuable for a wide range of environmental conditions and pollution types. For example, zero-valent iron (ZVI) nanoparticles have been exploited in treating hazardous and toxic heavy metals such as chromium, lead, or mercury, transforming them into their less toxic reduced forms.^4–6^ In addition, they have been used in advanced oxidation processes (AOPs) for the treatment of organic contaminants, taking advantage of the ability of ZVI nanoparticles as nanocatalysts to generate reactive oxygen species (ROS) in the presence of an oxidizing agent like hydrogen peroxide.^7–11^ ROS can partially or even completely mineralize many refractory organic pollutants into harmless carbon dioxide, water, and inorganic ions.^12^ Furthermore, ferromagnetic ZVI nanocatalysts exhibit very interesting magnetic properties, such as high saturation magnetization (*M*_S_) values,^13^ approximately twice as much as those of magnetite (another material known for its capacity to generate ROS),^14^ even at the nanoscale.^15^ These high *M*_S_ values allow for efficient removal through magnetic harvesting, which, compared with common separation technologies, results in a more cost-effective process with less energy consumption.

One of the main challenges that must be addressed when using ZVI nanocatalysts is their stabilization in colloidal suspension and protection against oxidation. When these nanocatalysts are in aqueous suspension, the formation of aggregates occurs due to interparticle forces such as dipolar magnetic interactions or van der Waals forces since these processes are thermodynamically favorable.^16^ The formation of these aggregates decreases the specific surface area of the material, making it less effective at trapping contaminants in solution. In addition, ZVI nanocatalysts are highly susceptible to oxidation due to their high surface area-to-volume ratio, which makes them more reactive and prone to interact with oxygen and other oxidizing agents in the environment. This leads to their deterioration over time, reducing their reusability.^17^ Therefore, stabilizing metallic ZVI nanocatalysts with protective coatings or stabilizers is nowadays the challenge to reduce oxidation and extend the nanocatalysts’ lifespan while preserving their properties over time.

Some of the most used stabilization methods to date involve the use of organic molecules such as polymers that coordinate to the surface of the nanoparticles,^18,19^ the formation of matrices and networks where the metallic iron nanoparticles are embedded,^20–22^ or the coating of these nanoparticles with a thin layer of inorganic compounds, by growing a thin layer of metal hydroxides on the surface of the nanoparticles.^23–25^ For example, Hu and Li demonstrated the stability of ZVI nanocatalysts for environmental remediation by using rate-controlled precipitation to form a protective thin layer of amorphous Al­(OH)_3_ onto them.^25^ Similarly, Maamoun et al. coated ZVI nanocatalysts with nonmagnetic layered hydroxides of Mg, Al, and Ca to enhance their stability.^26^ This is possible because of the reduction potential (Fe^2+^/Fe^0^ *E* ^°^ ≈ −0.76 V) exhibited by iron in the zero oxidation state,^27^ which allows for the surface reductive precipitation of metal ions present in suspension, forming their corresponding hydroxides under near neutral pH. Through this process, not only ionic species in solution can be removed (such as heavy metal cations), but these metals can also help passivating the surface of the nanoparticles, enabling the material to be used for a longer period. Coating the nanoparticles with magnetic elements such as Ni or Mn is expected to adjust their magnetic properties, such as coercivity (*H*_C_) and saturation magnetization (*M*_S_). Additionally, Mn has been reported to enhance ROS production,^28^ apart from providing protection against oxidation and degradation.

Electron paramagnetic resonance (EPR) has been used for identifying and quantifying ROS production from metal ferrites in the presence of hydrogen peroxide^29,30^ and it will be used in this work to determine the oxidation state of iron ions present in the sample, as well as to assess its potential for application in AOPs. The catalytic activity of ZVI nanocatalysts in AOPs is originated in the Fenton reaction when exposed to hydrogen peroxide (H_2_O_2_) at acidic pH. In the mentioned reaction, Fe^2+^ and Fe^3+^ ions degrade H_2_O_2_ forming hydroxyl (^•^OH) and hydroperoxyl (^•^OOH) radicals, respectively.^31–34^ These free radicals are highly reactive and can oxidize organic molecules, being ^•^OH the most active species.^35^

In our previous work, we defined the key parameters for synthesizing ZVI nanocatalysts using a strong alkaline polyol medium that allows the direct reduction of iron­(II) salts to iron(0) in the absence of any additional reducer.^36,37^ Combining surfactants and controlling the reaction temperature, pure cubic-shaped ZVI nanocatalysts smaller than 100 nm were obtained and stabilized against oxidation in polyol media. In this study, we further investigate the effect of various coatings, including polymeric and metal hydroxide coatings (M­(OH)_2_, M = Ni, Mn), on the stability of ZVI nanocatalysts in aqueous media and its effect on the ROS production, followed by EPR measurements. Aqueous media are used for applying nanoparticles in environmental remediation because contaminants, such as heavy metals and organic pollutants, are often found in water or soil with high moisture content. Additionally, the stability of the coating and the material’s resistance in acidic aqueous suspension against aging will be studied by analyzing the ROS generation kinetics. It has been demonstrated that magnetite nanoparticles (Fe^2+^Fe_2_^3+^O_4_) after continuous Fenton reaction transform into full oxidized maghemite (γ-Fe_2_^3+^O_3_),^38^ losing its activity. We expect that ZVI nanocatalysts properly protected will act as a source of electrons, regenerating Fe^2+^ ions at the surface and therefore extending the lifetime of the material and its functionality in AOP for longer. Coating magnetic nanoparticles with Mn and Ni is expected to enhance the magnetic properties and provide chemical and thermal stability.

Recent studies have highlighted the exceptional performance of iron-based nanomaterials in environmental applications. For instance, the integration of iron nanoparticles with natural additives such as *Aloe vera* biomass has shown significant improvements in biogas production during anaerobic digestion of waste sludge, demonstrating the synergistic effect of bio-based materials and iron catalysts in contaminant degradation processes.^39^ Moreover, the optimization of nZVI synthesis methods has proven effective for the removal of phosphorus and nitrate from aqueous solutions, emphasizing the importance of cost-efficiency and parametric control in material preparation.^40^ Additionally, the chemical deposition of Fe^0^ nanoparticles on titanium nanowires has enabled efficient adsorption of pharmaceutical contaminants like ciprofloxacin, showcasing the versatility of iron-based systems in water purification technologies.^41^ These findings reinforce the relevance of understanding both the structural characteristics and the mechanistic pathways of contaminant removal, which are crucial for designing advanced materials tailored to specific environmental challenges.

Building on these advances, this study aims to systematically evaluate the effect of polymeric and inorganic coatings on the stability and catalytic performance of ZVI nanocatalysts in aqueous media. By analyzing ROS generation kinetics through EPR and assessing structural integrity via transmission electron microscopy (TEM) and magnetic measurements, we seek to identify coating strategies that enhance the resistance to oxidation and sustain catalytic activity under acidic conditions. This approach provides a comprehensive understanding of how surface engineering influences the long-term functionality of ZVI nanocatalysts in Fenton-like reactions.

## 2 Experimental Section

### 2.1 Chemical Reagents

Reagents, such as iron­(II) chloride (FeCl_2_, ≥99%), aluminum chloride (AlCl_3_, ≥99%), nickel chloride (NiCl_2_, ≥99%), manganese chloride (MnCl_2_, ≥99%), sodium hydroxide (NaOH, ≥99%), ethylene glycol (EG, ≥99%), diethylene glycol (DEG, 99%), hydrogen peroxide (H_2_O_2_, 30%), d-mannitol 99%, PVP (*M*_w_ 40.000), methanol, and ethanol, were purchased from Sigma-Aldrich. 5,5-dimethyl-1-pyrroline *N*-oxide (DMPO, 98%) was purchased from Cayman Chemicals, with the exception of hydrogen peroxide all the reagents and solvents were in anhydrous form.

### 2.2 Synthesis of Coated Zero-Valent Iron Nanocatalysts

ZVI nanocatalysts were synthesized following the methodology described in our previous work^36,37^ where the reaction times and conditions are thoroughly detailed and optimized for reproducibility. In a typical experiment, FeCl_2_, d-mannitol, and PVP were added in a 1:1:1 mass ratio in 100 mL of ethylene glycol (EG) with a molar ratio FeCl_2_/EG = 0.005. The solution was heated to 175 °C at 4 °C/min under nitrogen bubbling for 30 min to remove water from the system. Subsequently, solid NaOH pellets were added (mass ratio FeCl_2_/NaOH = 0.025, equivalent to 9 g of NaOH). Finally, the black precipitate was separated magnetically or by centrifugation and washed with anhydrous methanol to remove NaOH excess, byproducts, and unbound surfactants. This sample was labeled as ZVI@MP (zero-valence iron nanoparticles coated with mannitol and PVP).

Further protection was achieved by coating the ZVI@MP with metal hydroxides (Mn­(OH)_2_ and Ni­(OH)_2_) following a previously described protocol with slight modifications,^42^ resulting in the samples labeled as ZVI@MP@Mn and ZVI@MP@Ni. Specifically, 40 mL of ethanol containing 2 mg/mL ZVI@MP were sonicated for 10 min under a nitrogen atmosphere in a closed vial. Subsequently, 9 mL of another ethanol solution containing 1 mg/mL either MnCl_2_ or NiCl_2_ was added to the nanocatalysts’ solution. Finally, 3 mL of 1 mg/mL NaOH in ethanol was prepared and added to the mixture at a rate of 1 mL/min under sonication. This final step ensured the formation of a thin protective layer of metal hydroxides on the ZVI nanocatalysts.

Finally, the ZVI@P1 and ZVI@P2 samples were obtained using PVP as the sole stabilizing agent, following the same synthesis procedure described for ZVI@MP but without adding d-mannitol. The stabilization was achieved by varying the PVP mass ratio relative to FeCl_2_. ZVI@P1 was prepared with a 1:1 FeCl_2_/PVP mass ratio, while ZVI@P2 was obtained with a 1:2 FeCl_2_/PVP mass ratio. These samples were designed to explore the effect of increasing the PVP content on the structural stability and magnetic properties of the nanocatalysts.

Figure 4 includes a schematic representation of the core–shell structure of the coated ZVI nanocubes, illustrating the metallic iron core and the protective polymer/metal hydroxide layers applied during synthesis.

**Figure 1:**
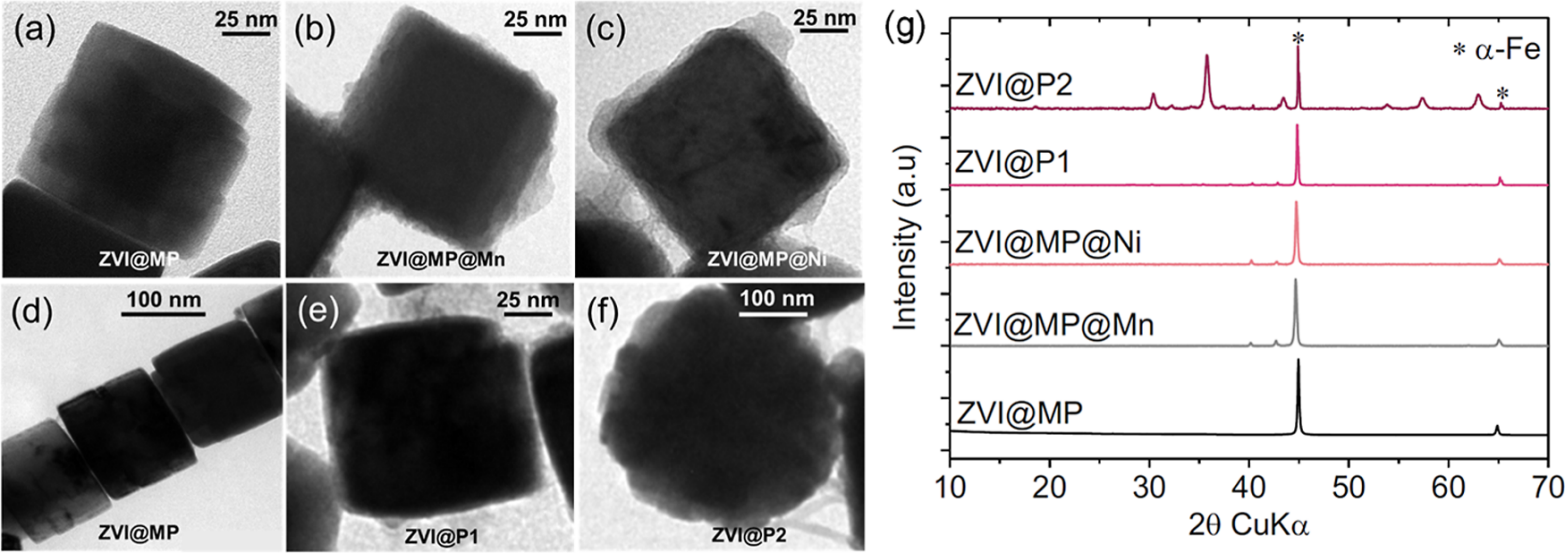
TEM micrographs of ZVI@MP (a), ZVI@MP@Mn (b), ZVI@MP@Ni (c), ZVI@P1 (e), and ZVI@P2 (f). TEM micrographs of ZVI@MP forming chains (d). XRD pattern of all the samples (g). α-Fe peaks are marked with (*).

**Figure 2:**
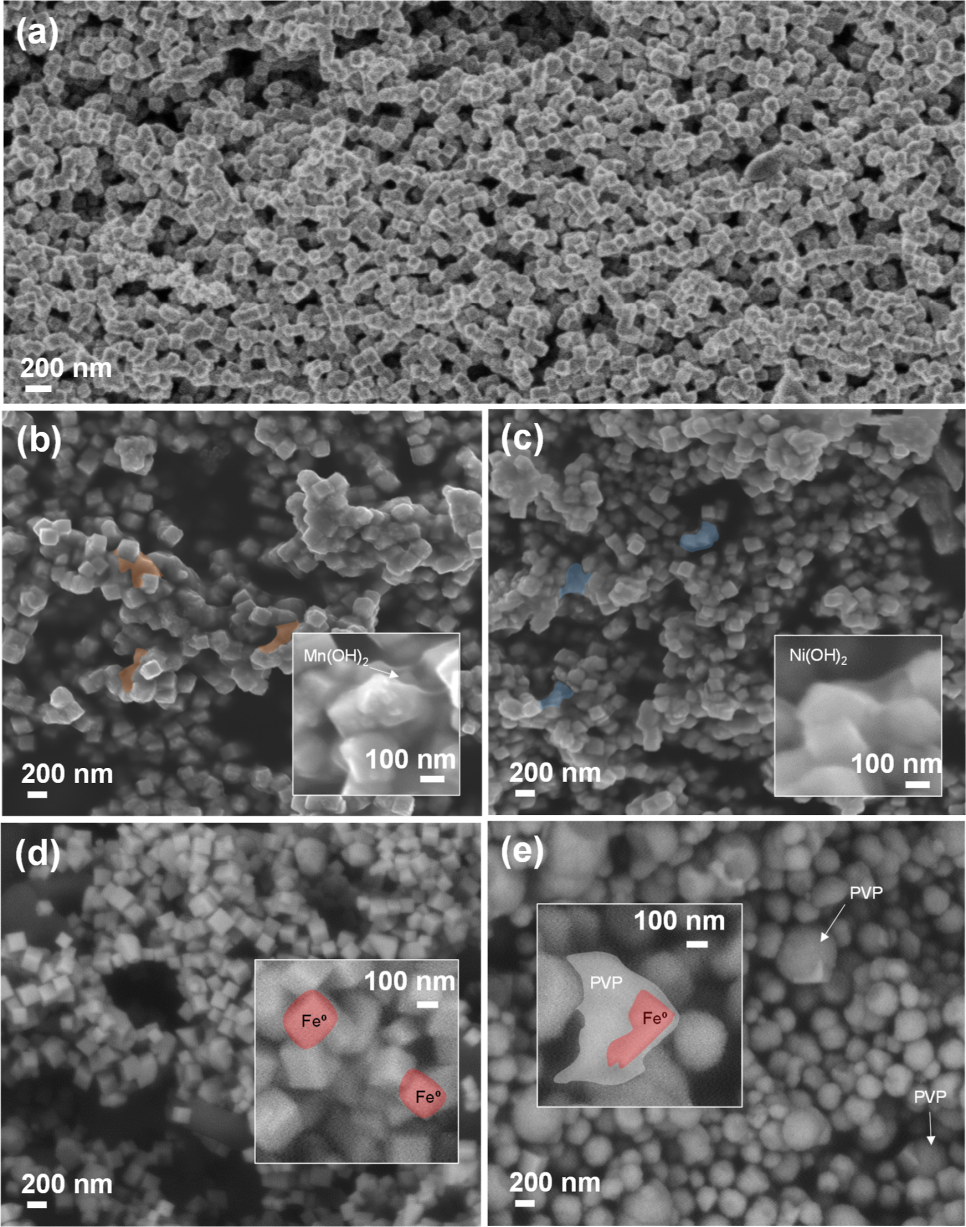
SEM micrographs of ZVI@MP (a), ZVI@MP@Mn (b), ZVI@MP@Ni (c), ZVI@P1 (d), and ZVI@P2 (e). The Mn coating is marked in orange (**2b**), and the Ni coating is marked in blue (**2c**). The insets show images at higher magnifications.

**Figure 3:**
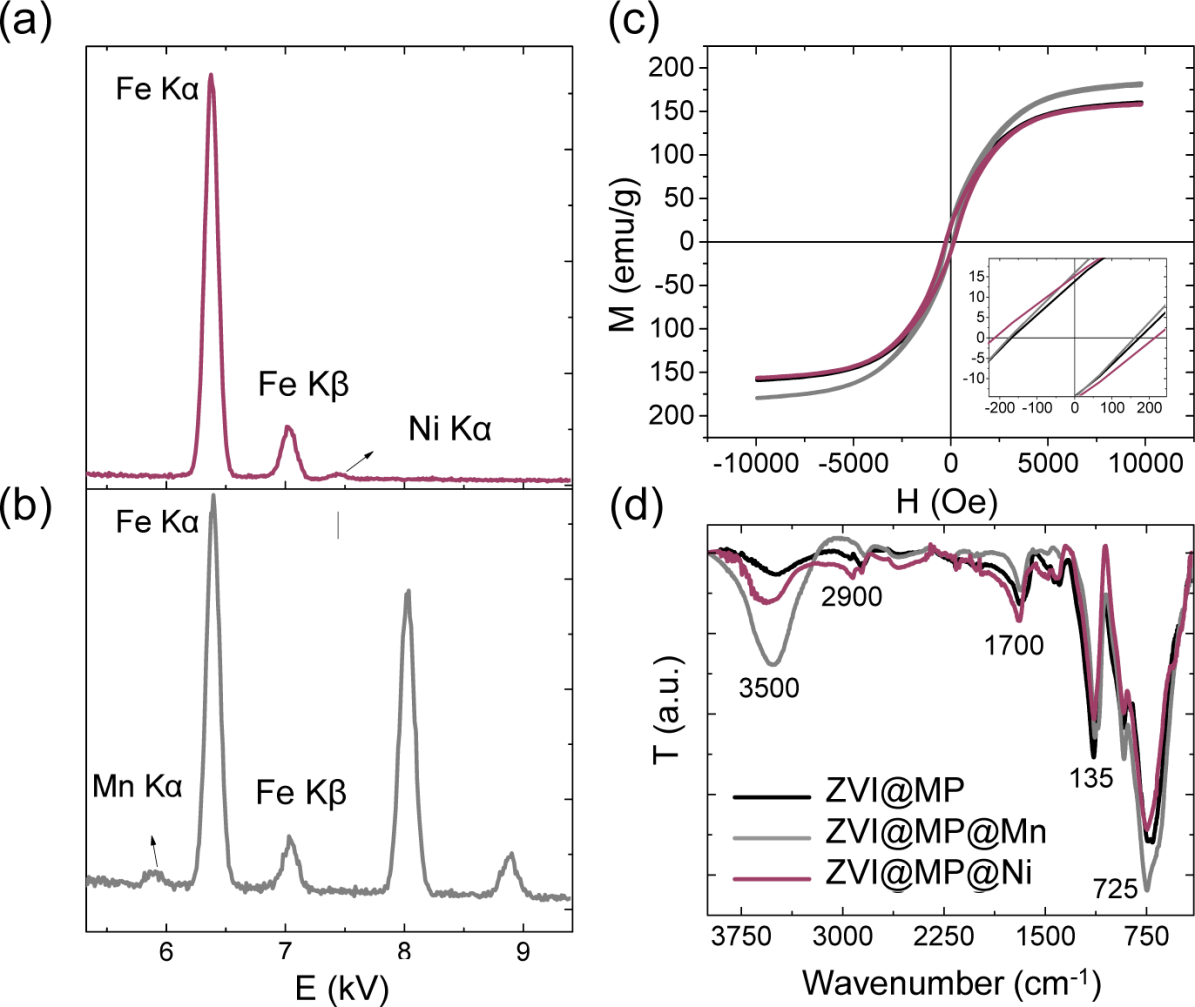
Elemental, surface, and magnetic characterization of inorganic-coated ZVI nanocatalysts. Energy-dispersive spectroscopy (EDX) analysis of ZVI@MP@Ni (a) and ZVI@MP@Mn (b). Magnetization hysteresis loops at room temperature (c) of ZVI@MP (black), ZVI@MP@Mn (gray), and ZVI@MP@Ni (red), and infrared spectra of samples (d).

**Figure 4:**
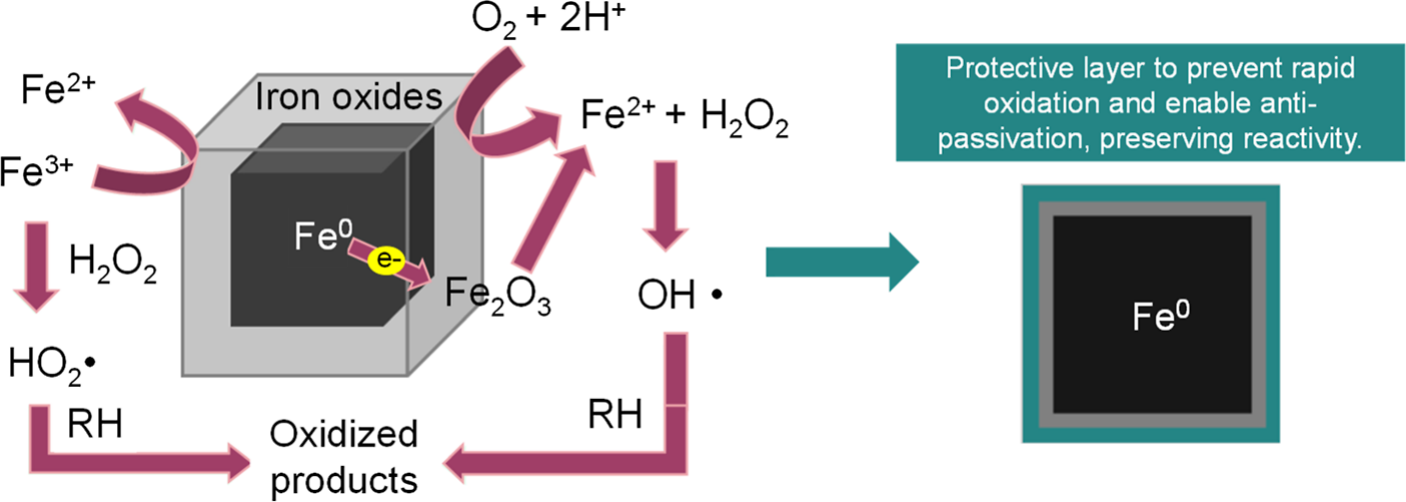
Schematic representation of the core–shell structure formation, surface oxidation mechanism, and protective layer function in ZVI nanocatalysts. Antipassivation prevents surface passivation, maintaining electron transfer and Fe^0^ reactivity.

### 2.3 Structural and Magnetic Characterization Techniques

The particle size, shape, and distribution were determined by TEM in a JEOL JEM 1010 (Pleasanton, CA, USA) operated at 100 kV. Sample preparation involves placing a drop of the diluted samples onto a copper grid coated with amorphous carbon, allowing it to dry completely before observation. To obtain the mean particle size and distribution, at least 250 nanoparticles were measured and analyzed by using ImageJ software. The obtained histograms were fitted to a Gaussian curve to obtain the average size and standard deviation. Scanning electron microscopy (SEM) in a NOVA NANO SEM 230 instrument was performed to characterize the sample surface. For that analysis, a diluted suspension of ZVI nanocatalysts in ethanol was deposited onto an amorphous, carbon-coated copper grid. Through energy-dispersive X-ray spectroscopy (EDX), it was possible to quantify the percentage of transition metal present in the samples coated with hydroxides, following the same sample preparation procedure as for SEM. Crystalline structure of the powder samples was determined by standard X-ray diffraction (XRD) using a Bruker D8 Advance diffractometer with a graphite monochromator using Cu Kα radiation (λ = 1.5406 A), within 5 and 70 2θ degrees. Crystal size was determined by considering the full width at half-maximum of the (110) α-Fe peak and using the Scherrer formula. A LakeShore 7300 vibrating sample magnetometer was used to measure the magnetic properties of the particles. The preparation of the sample consisted of putting some drops of an ethanol suspension of the ZVI nanocatalysts on a piece of cotton. After being dried, the samples were pressed into the sample holder. The hysteresis loops of the powder samples were measured at 290 K up to ±10,000 Oe, and the *M*_S_, given by emu/g of nanocatalyst (containing organic and inorganic coatings), was obtained by extrapolating to 1/H = 0 the high-field part of the magnetization curve.

All measurements were performed using calibrated instruments, and the reported values reflect the precision and accuracy specified by the equipment manufacturers. Instrumental uncertainties were considered during data acquisition and presentation, ensuring the reliability of the reported results within the constraints of the study.

### 2.4 Identification and Quantification of Reactive Oxygen Species

EPR measurements were performed to evaluate the Fenton activity and stability of the prepared samples by employing the standard spin-trap method. The Fenton activity was tested in a solution containing 100 μg of ZVI in 100 μL of an acetate buffer solution (0.1 M, pH = 5) and 25 μL of a solution 1 mg/6 mL of the standard spin-trap 5,5-dimethyl-1-pyrroline *N*-oxide (DMPO) in dimethyl sulfoxide (DMSO). To start the Fenton reaction, 5 μL of hydrogen peroxide was added to the solution. The addition of H_2_O_2_ marked the initial time of the reaction, which was monitored for 1 h by collecting spectra at 5 min intervals considering different aged samples (0, 1, 5, and 7 d). For the stability assessment, samples were stored in solution at different pH values (4, 7 and 10) for 24 h. The capability of the ZVI particles for maintaining the production of Fenton radicals was tested by repetition of the previous analysis at different time intervals.

The EPR measurements were performed in a Bruker ELEXSYS II-E500 spectrometer equipped with an X-band resonant cavity operating at 9.4 GHz. Spectra were collected under controlled conditions at 20 ± 1 °C, with a modulation signal of 100 kHz and 3 Oe of amplitude. The EPR spectra were compared with the signal of pattern sample of MgO/Mn^2+^ to normalize the free radical production in each sample. This technique allows the detection of species with unpaired electrons, making it a powerful technique for identifying short-lived radicals. In this study, ROS generation was monitored using 5,5-dimethyl-1-pyrroline-*N*-oxide (DMPO) as a spin-trapping agent. DMPO reacts with hydroxyl radicals to form a stable DMPO–OH adduct, which exhibits a characteristic quartet signal in the EPR spectrum. The intensity of this signal correlates with the concentration of ^•^OH radicals, allowing a quantitative comparison of ROS generation under different experimental conditions.

All EPR experiments were conducted at room temperature (RT, 20 ± 1 °C). This temperature was selected based on previous observations indicating that metallic iron nanoparticles degrade more rapidly at elevated temperatures in aqueous media. Performing the tests at RT ensured the stability of the ZVI nanocatalysts during ROS production measurements and allowed for a consistent comparison across different coating conditions. The nanocatalysts were suspended in acetate buffer at pH 5 for stability and ROS generation tests because pH 5 is widely reported in the literature as the optimal condition for Fenton-like reactions. At this pH, Fe^2+^ ions are sufficiently stable and reactive to catalyze the decomposition of hydrogen peroxide into hydroxyl radicals (^•^OH), which are the most potent reactive oxygen species for pollutant degradation.

## 3 Results

### 3.1 Protected Zero-Valent Iron Nanocatalysts

The ZVI nanocatalysts prepared by the polyol process in the presence of PVP and mannitol (ZVI@MP) can be described by its nanometer size (80 ± 20 nm), its homogeneity (<30% of polydispersity degree), and a marked cubic shape as revealed by TEM images (Figure 1a). The cubic morphology of ZVI nanocatalysts is attributed to the polyol synthesis method under strong alkaline conditions, which favors anisotropic growth, as previously demonstrated for metallic iron nanoparticles. Inorganic coating of Mn (Figure 1b) and Ni hydroxides (Figure 1c) on ZVI@MP nanocatalysts, coated in a second step by direct precipitation, appears as irregular deposits (lower contrast) on the cubic ZVI nanocatalysts (higher contrast) (samples ZVI@MP@Mn, and ZVI@MP@Ni). XRD patterns (Figure 1g) before and after coating show only the peaks (110) and (200) corresponding to the α-Fe structure (JCPDS No. 65-4899). Crystal sizes of all samples compared with the TEM sizes are shown in Table S1. In all cases, the crystal size of the nanocatalyst is smaller than the TEM size, demonstrating that the particles are composed of several misoriented crystalline domains.

The ZVI nanocatalysts coated with organic material were synthesized by introducing only PVP in a single-step process (ZVI@P1 and ZVI@P2). This method resulted in larger metallic iron cores of 153 ± 45 nm, which appeared with higher contrast under electron microscopy due to their greater electron density, surrounded by a lower-density organic matrix (Figure 1e). A further increase in PVP leads to the formation of particles with increased particle size and spherical morphology (249 ± 85 nm), most probably consisting on PVP spheres containing the ZVI nanocatalysts (Figure 1f), as previously shown for copper particles encapsulated in PVP.^43^ TEM images of the samples at low magnification together mean size and particle size distributions for all samples are presented in the Supporting Information (Figure S2). For the ZVI@P2 sample, the diffraction pattern reveals the secondary presence of an iron oxide spinel structure (JCPDS No. 19-0629) (Figure 1g).

Coated nanocatalysts were further analyzed by SEM (Figure 2). Figure 2a shows a uniform size distribution of the monodisperse synthesized ZVI@MP nanocatalysts, highlighting their well-defined cubic shape with sizes below 100 nm. Figure 2b,c validates the presence of a Mn coating in orange (Figure 2b) and of the Ni coating in blue (Figure 2c) identified as a veil covering the cubes. Magnified insets in both micrographs show the presence of less dense hydroxide layers covering the ZVI nanocubes. Although the coatings applied to the ZVI nanocatalysts were not uniformly distributed across all particles, achieving uniform coating could further enhance the performance and stability of the system. A homogeneous passivation layer would ensure consistent protection against oxidation, reduce variability in ROS generation, and improve colloidal stability. Moreover, uniform coating may facilitate better control over catalytic activity and magnetic properties, which are critical for reproducibility and scalability in environmental applications.

The EDX analysis confirmed the presence of Mn and Ni on the Fe nanocubes (Figure 3a,b). A peak corresponding to the Mn Kα band is observed at energies near 6 kV for ZVI@MP@Mn, while the Ni K-alpha band appearing at 7.5 kV is observed for ZVI@MP@Ni. Furthermore, both spectra exhibit the K-alpha and K-beta bands of Fe stemming from the ZVI cores and two peaks close to 8 and 9 kV that correspond to Cu from the grid support. Infrared spectroscopy in Figure 3d reveals an enhancement of the band around 3400 cm^–1^ corresponding to the vibration of OH groups from the Mn and Ni and confirms the hydroxylic nature of the coatings. Additionally, common features across all three samples (ZVI@MP, ZVI@MP@Mn, and ZVI@MP@Ni) include bands at 1461, 1117, 1051, and 869 cm^–1^, indicative of C–O and C–C stretching and C–C connections (in the aliphatic backbone and pyrrolidone ring) characteristic of d-mannitol and PVP at the nanoparticle surface.

Organic-coated nanocatalysts under the SEM are depicted in Figure 2d,e using only PVP at two concentrations. The SEM image of the ZVI@P1 sample (Figure 2d) aligns with the TEM image (Figure 1e), revealing a distribution of cubes along with remnants of PVP between particles. As illustrated by SEM, doubling the PVP concentration leads to the formation of PVP spheres that encapsulate the ZVI nanocubes, providing protection for future applications (Figure 2e).

The magnetization curves in Figure 3c demonstrate a ferromagnetic behavior at room temperature for all samples, consistent with the nanoparticle sizes larger than 20 nm, exhibiting saturation magnetization of 156 emu/g for ZVI@MP and 155 emu/g for ZVI@MP@Ni, and slightly larger saturation magnetization of 175 emu/g for ZVI@MP@Mn. The reduction in magnetization compared to bulk iron (218 emu/g at room temperature) is due to both, the surfactants employed in the synthesis (e.g., 12.5% of organic matter observed in previous work for sample ZVI@MP^36^), and to the presence of the oxide layer, which seems to be thinner for the Mn hydroxide-coated samples. Similar results were found for Al and Ca hydroxide-coated iron nanoparticles, with an important reduction in *M*_S_ when increasing the oxide content.^44^ It seems that the Mn hydroxide layer acts as a good passivating agent for the ZVI nanocubes, effectively preventing further oxidation and *M*_S_ reduction. Moreover, the samples display coercive field values of 182, 221, and 166 Oe, for ZVI@MP, ZVI@MP@Ni, and ZVI@MP@Mn, respectively, similar to what have been reported for iron metal nanoparticles with sizes between 20 and 100 nm, having coercivity usually between 100 and 1000 Oe, depending on the axial ratio.^45–47^

Magnetization curves for the organic-coated samples are shown in Figure 5. *M*_S_ values of 107 and 99 emu/g were obtained for samples ZVI@P1 and ZVI@P2 respectively, significantly lower than the *M*_S_ value for the inorganic-coated particles, indicating that over 50% of the sample weight consists of polymer and oxide. The presence of polymeric material was followed by TGA under an air atmosphere (Figure S3), although the results do not permit an accurate estimation of the material quantity due to the superposition of the mass loss, corresponding to the degradation of PVP, with the increase in mass due to the oxidation of the metal iron core at elevated temperatures. Moreover, there is a significant decrease in *H*_C_ for ZVI@P1 (104 Oe) and furthermore for ZVI@P2 sample (32 Oe), as observed in Table 1, which could be attributed also to the reduction in magnetic interactions, avoiding the formation of chains, clear from SEM images in Figure 2.

**Figure 5:**
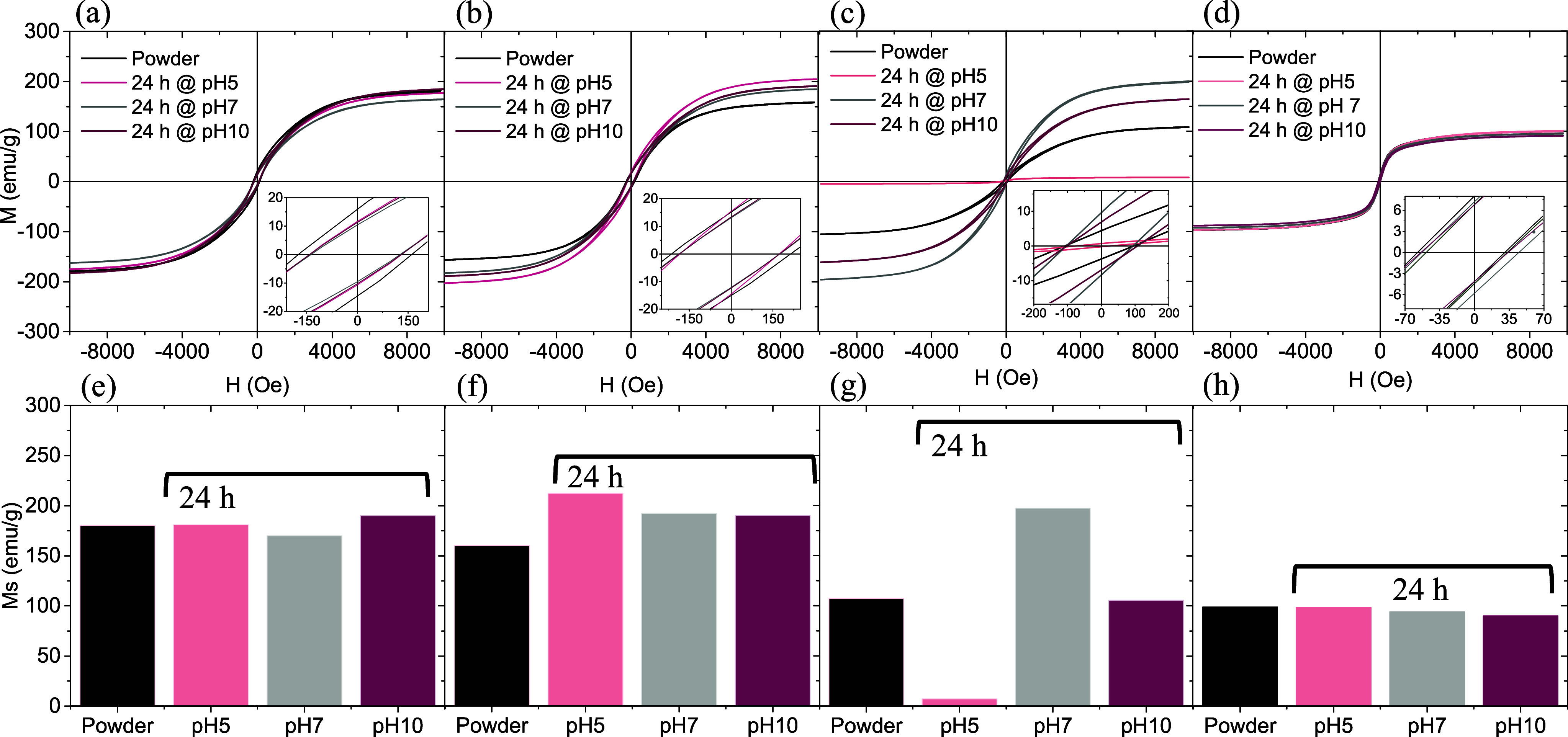
Magnetization hysteresis loops at room temperature of ZVI@MP@Mn (a), ZVI@MP@Ni (b), ZVI@P1 (c), and ZVI@P2 (d). Magnetization saturation values of ZVI@MP@Mn (e), ZVI@MP@Ni (f), ZVI@P1 (g), and ZVI@P2 (h) measured in powder and after suspension in water at different pH (5, 7 and 10) after 24 h.

**Table 1:** Magnetic Parameters of Coated ZVI Nanocatalysts at Room Temperature

sample (−)	time in buffer (h)	pH (−)	*M*_S_ (emu/g)	*M*_R_/*M*_S_ (emu/g)	*H*_C_ (Oe)
ZVI@MP	0	-	158.3	0.09	182
	0	-	179.5	0.09	167
	24	5	176.6	0.06	135
ZVI@MP@Mn	24	7	181.7	0.06	129
	24	10	184.8	0.06	138
	0	-	156.7	0.09	222
	24	5	205.1	0.07	183
ZVI@MP@Ni	24	7	183.2	0.07	181
	24	10	191.6	0.09	180
	0	-	107.5	0.04	104
	24	5	7.3	0.14	58
ZVI@P1	24	7	197.5	0.05	100
	24	10	165.7	0.04	115
	0	-	98.9	0.08	32
	24	5	98.5	0.06	35
ZVI@P2	24	7	94.2	0.07	46
	24	10	90.1	0.07	36

Remanent magnetization is in all cases very low, much lower than the 0.5 value reported for a random array of single domain particles.^48,49^ Considering that the size of the particles prepared in this work is far from being in the superparamagnetic range, the low *M*_R_ together with the low *H*_C_ indicates a multidomain character with the magnetization rotation taking place via wall motion. In the multidomain state, these domains can reorient under an external magnetic field, but once the field is removed, they may not fully realign, leading to a low residual remanent magnetization. Further analysis is required to confirm the multidomain character of the samples, analyzing if the remanence is unchanged with cooling and the coercivity does not depend on packing fraction. Hysteresis loops at 5 K are shown in Figure S4, and the remanence and coercivity data are collected in Table S2. It is observed that remanence values do not change with cooling, while coercivity values go from 140 to 175 Oe for ZVI@MP@Mn and from 185 to 215 Oe for ZVI@MP@Ni. Coercivity is difficult to analyze since due to the high magnetic moment per particle, particles spontaneously join forming chains (Figure 1d), affecting the *H*_C_ value. It should emphasize the interest in the magnetic behavior of these samples, particularly its high saturation magnetization values, low coercivity, and low remanence, for magnetic separation applications in water treatment, where particles need to remain magnetically responsive but not retain magnetization when the external field is removed.

### 3.2 Stability of ZVI Nanocatalyst Suspensions

To evaluate the chemical stability of ZVI nanocatalysts in water suspensions at different pH levels, magnetization curves of the coated samples were measured after 24 h of storage in solutions with pH 5, pH 7, and pH 10 at room temperature. All measurements, shown in Figure 5, were taken employing suspensions prepared with dry powder of the nanocatalysts, and the results were compared to the magnetization curves of the initial, untreated samples (referred to as “powder”). As observed in Figure 5, when the coating on ZVI nanocatalysts remains stable, no significant changes in saturation magnetization values are evident after 24 h of storage at various pH levels. This stability is demonstrated in nanocatalysts coated with manganese hydroxide, as shown in Figure 5a,e. Conversely, when the coating is unstable, as with the Ni­(OH)_2_ coating, notable variations in the *M*_S_ values occur. For instance, Figure 5b,f indicates an increase in magnetization after suspension in water at different pH levels compared with the fresh samples. This increase is attributed to the partial dissolution of nonmagnetic components, such as the Ni­(OH)_2_ or PVP coating. Since the magnetization is normalized per gram of material, the *M*_S_ value increases because the magnetic content with respect to the nonmagnetic one increases.

Furthermore, in Figure 4, a schematic representation illustrates the process by which an oxide layer forms on the surface of metallic iron cubes. This surface layer plays a key role in the Fenton-like reaction, acting as a catalytic interface where hydrogen peroxide interacts with the iron surface to generate reactive oxygen species. In these systems, the coating applied to the ZVI cubes acts as an antipassivation layer, preventing the development of dense, compact oxides that would otherwise passivate the surface. By inhibiting rapid passivation, the coating preserves the catalytic activity of the Fe^0^ core throughout the AOP process. The diagram highlights how the transformation of Fe^0^ to Fe^2+^ and subsequently to Fe^3+^ contributes to the continuous production of radicals, thereby enhancing the oxidative capacity of the nanocatalysts.

A high-resolution TEM image of the untreated ZVI sample is provided in Figure S1 of the Supporting Information, showing a thin surface oxide layer of a few nanometers surrounding the iron nanocubes. This observation is consistent with previous studies that reported core–shell structures in ZVI nanoparticles using techniques such as X-ray photoelectron spectroscopy and Mössbauer spectroscopy.^50,51^ In our case, the presence and thickness of this oxide layer vary depending on the type of surface coating and the pH conditions, which directly influence the extent of iron oxidation and, consequently, the magnetic and catalytic behaviors of the material.

For the organic PVP coatings, it should be mentioned that, as revealed by SEM (Figure 2d inset), the ZVI@P2 sample exhibits nearly complete coverage of ZVI cubes by a PVP sphere, forming a core–shell architecture, which contributes to the lower saturation magnetization compared to ZVI@P1. Additionally, the presence of iron oxide passivating the surface of the nanoparticles, as observed in the XRD pattern (Figure 1g) despite the higher PVP content, also plays a significant role in the decrease in saturation magnetization. However, this double surface layer made of iron oxide and PVP forms a protective microstructure that effectively shields the iron cores from further oxidation (Figure 5d).

As a result, the magnetic properties of the ZVI@P2 sample show minimal variation at different pH values, with a slight decrease in magnetization observed at neutral and alkaline pH values (Figure 5d, h). Furthermore, ZVI@P2 shows the lowest coercivity among the samples, which can be attributed to its multidomain magnetic structure. On the other hand, following exposure to pH 5 for 24 h, the ZVI@P1 sample displayed an important reduction in magnetization due to near-complete nanocube dissolution in the acidic environment. In contrast, at pH 7 and 10, saturation magnetization values resemble those of bulk iron, indicating that the iron core is not affected but the residual PVP adhered to the particles has been dissolved. It is understood that PVP, owing to its functional groups, exhibits higher solubility in neutral or basic environments.^52^ This suggests that the reduction of *M*_S_ that results from treatment at pH 10 with respect to that obtained at pH 7 for this sample is due to the oxidation of the metal cores. The general conclusion about ZVI@P1 is that the sample was not protected enough by PVP coating, and its behavior is a consequence of the dissolution of PVP and/or the dissolution of the magnetic cores. Table 1 displays the saturation magnetization, coercive field, and remanence (*M*_R_) values of the samples under various conditions, as measured from the magnetization curves depicted in Figure 5. It is important to highlight that, in both samples coated with metal hydroxides, the values of *H*_C_ and *M*_R_ remain stable after being suspended for 24 h, regardless of pH. Only in the ZVI@MP@Ni sample, there is a significant increase in *M*_R_ by 30% (from 14 to 18 emu/g) after suspension at pH 10, which, along with an increase in saturation magnetization (*M*_S_), could indicate a loss of the coating.

As mentioned before, all samples exhibit low *M*_R_/*M*_S_, both before and after being suspended in water at different pH levels. The most notable change occurs in the ZVI@P1 sample at pH 5, where there is a drastic decrease in *M*_R_/*M*_S_ due to partial dissolution of the sample at that pH. On the other hand, the ZVI@P2 sample maintains its *M*_R_/*M*_S_ and *H*_C_ values intact after being dispersed in water, regardless of pH, indicating the effectiveness of the coating. Table 1 shows the values of *M*_S_, *M*_R_/*M*_S_, and *H*_C_ obtained for all of the samples before and after being suspended in water at different pH levels.

### 3.3 Identification and Quantification of Reactive Oxygen Species

EPR allows for the identification and quantification of free radicals and species with unpaired electrons.^53^ Here, we will analyze the ROS generated by the ZVI nanocatalysts with different coatings through a Fenton-like process, i.e., in the Fe^2+^/H_2_O_2_ system. It is widely recognized in the literature on Fenton-like catalytic processes that the types of reactive oxygen species generated depend on the oxidation state of the iron species involved.^12^ Specifically, when the process is mediated by Fe^2+^, the predominant radical formed in the reaction with hydrogen peroxide is hydroxyl radical (^•^OH).^54^ In contrast, when Fe^3+^ is responsible for radical formation, hydroperoxyl radical (^•^OOH) is obtained,^28^ which indicate the boundary of the applicability of the Fenton reaction due to the lower formation kinetic constant. Figure 6 presents the deconvoluted EPR spectra of the ZVI@MP sample, highlighting the presence of three distinct paramagnetic species: (1) ^•^OH radicals produced from Fe^2+^, (2) ^•^CH_3_ radicals, likely formed through secondary reactions between DMPO and acetate molecules from the buffer, and (3) ^•^N radicals.

**Figure 6:**
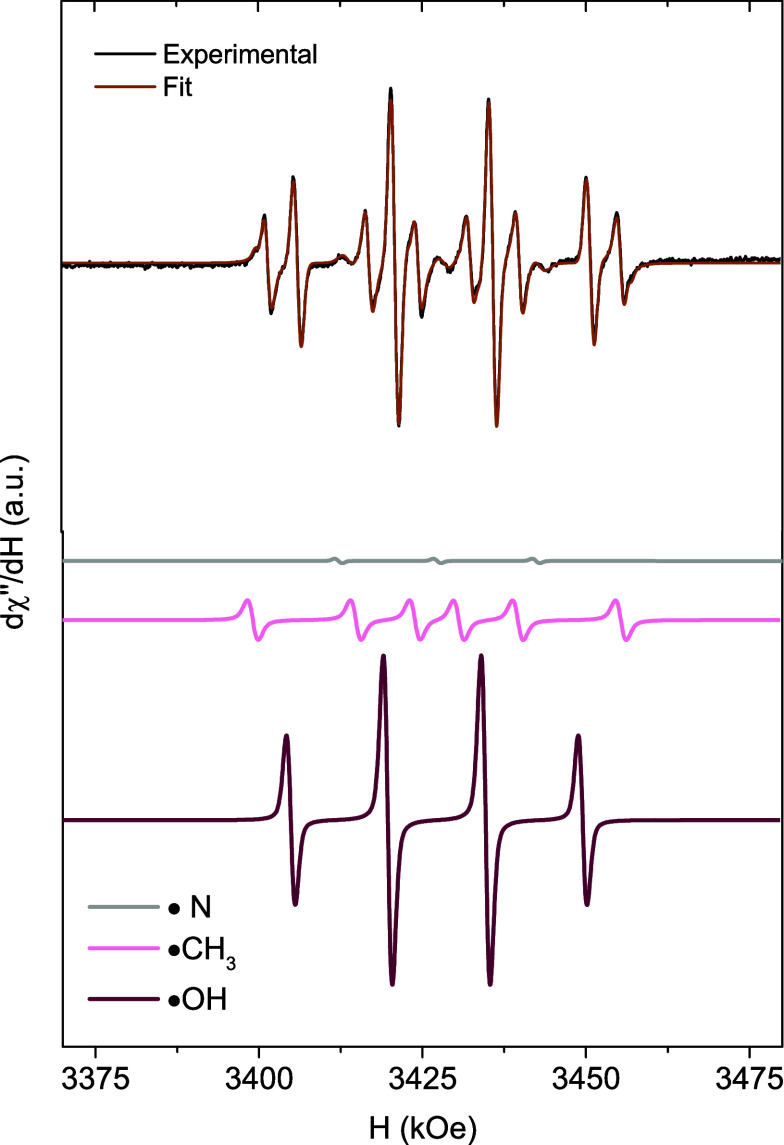
Deconvoluted EPR spectrum and corresponding fitting for the ZVI@MP sample in DMPO/DMSO, measured 5 min after the addition of H_2_O_2_ at room temperature at pH 5. The fitted spectra include the DMPO-adduct radical components.

To further investigate the formation of free radicals, the corresponding areas of each DMPO-adduct can be quantified from the EPR spectra. As anticipated for ZVI nanocatalysts, the signal associated with ^•^OH radicals exhibits the largest areas, reflecting the abundance of Fe^2+^ in the sample. This confirms that the ZVI core functions as an electron pump, continuously regenerating Fe^2+^ at the surface. To provide a consistent basis for comparison, all EPR spectra were normalized by using a MgO/Mn^2+^ reference, allowing relative quantification of the DMPO-adduct signals. This normalization confirms the differences in ROS generation among coatings, with ^•^OH radicals being predominant as expected for ZVI-based systems. The estimated experimental uncertainty in ROS determination is within ±10%, based on the sensitivity of the Bruker ELEXSYS II-E500 spectrometer and the normalization procedure.

Figure 7 illustrates the kinetics of ROS generation at different aging times for the inorganic-coated nanocatalysts (Figure 7a–c) suspended in acetate buffer (pH 5). The species identified as ^•^OH originate in the Fenton-like reaction, while the methyl radical (^•^CH_3_) is a product of a secondary reaction between ^•^OH and organic matter probably from the acetate buffer. As observed in Figure 7a–c, at 0 h in buffer, at initial time immediately after the addition of hydrogen peroxide, all three samples present an elevated production of ^•^OH radicals immediately after the addition of hydrogen peroxide, followed by a decline to near-zero levels after 1 h. At this time point, the samples exhibiting the highest radical production are ZVI@MP@Ni and ZVI@MP.

**Figure 7:**
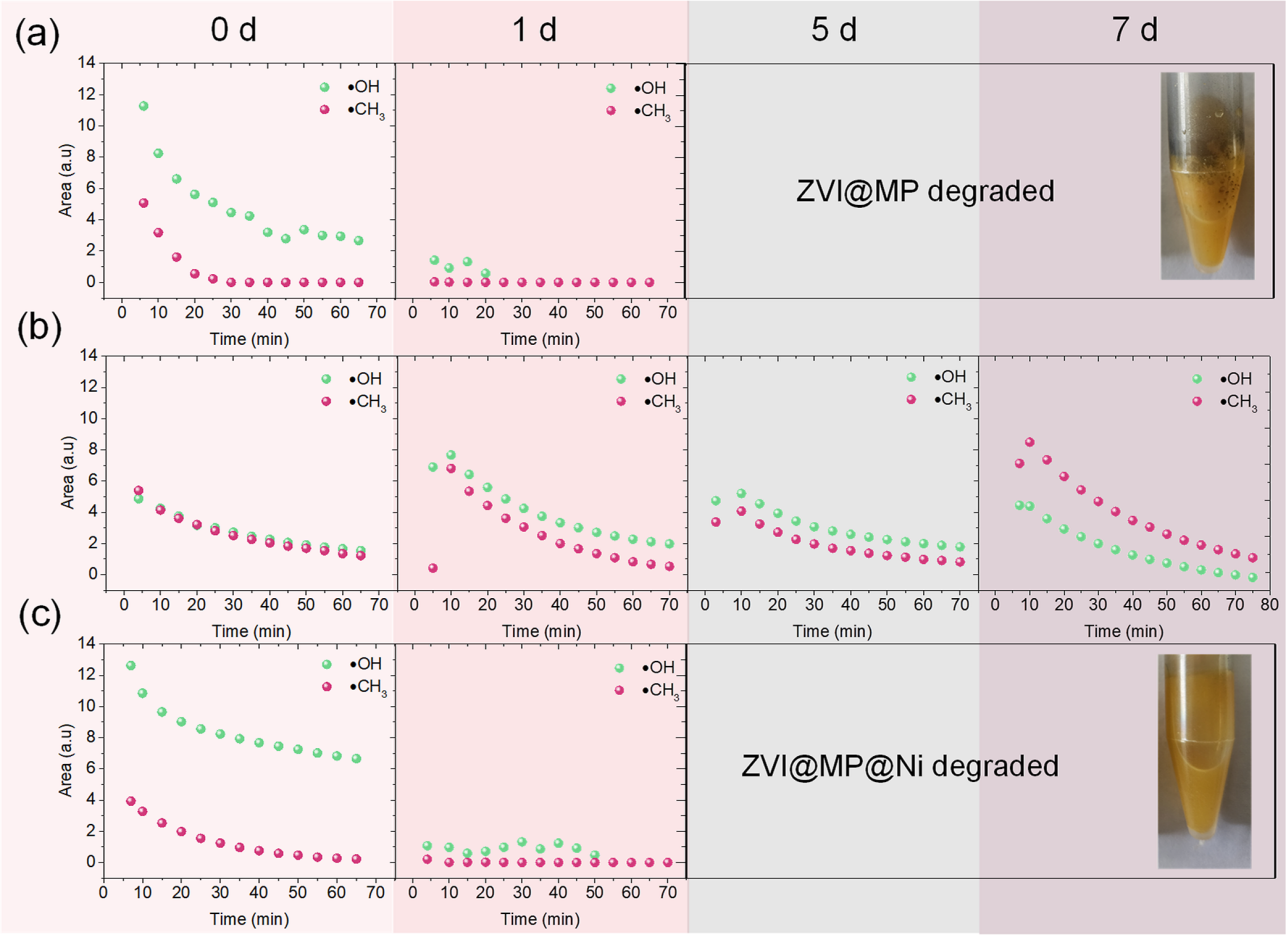
Kinetic curves of the reactive oxygen species catalyzed by ZVI@MP (a), ZVI@MP@Mn (b), and ZVI@MP@Ni (c) measured at different aging times (0, 1, 5, and 7 d) in buffer at pH 5. All EPR measurements were normalized using a MgO/Mn^2+^ reference, and the estimated experimental uncertainty in ROS determination is within ±10%.

After 20 min, the ZVI@MP sample shows a rapid decrease in reactivity, producing only 10% of ^•^OH radicals, while ZVI@MP@Ni still keeps 50% of the radical production in the same time frame. This indicates faster oxidation of the Ni­(OH)_2_ layer in the sustained production of ^•^OH radicals during the first hour of the process. After 1 d and beyond, both ZVI@MP and ZVI@MP@Ni nanocatalysts ceased to produce ^•^OH radicals due to the significant degradation resulting from storage in acidic media, which suffered complete degradation after 5 d.

In contrast, the ZVI@MP@Mn sample showed a lower but sustained production of radicals for 1 week in acetate buffer. The observed increase in radical production after 1 d of storing may be attributed to a partial loss of the hydroxide layer, which exposed a higher proportion of Fe^2+^ ions capable of generating ^•^OH. However, the progressive loss of the hydroxide layer subsequently rendered the material less protected, leading to increased deterioration over time and a subsequent decrease in ^•^OH radical production beyond the 24 h-mark, but not reaching full degradation of the material in the time-window of the experiment.

The different behavior observed between ZVI@MP@Mn and ZVI@MP@Ni samples can be attributed to the different acidity conditions under which they are stored. While both hydroxides should theoretically dissolve more easily at acidic pH, the oxidation-prone nature of Mn^2+^ could lead to precipitation of MnO_2_ or other insoluble manganese oxides. This makes the Mn­(OH)_2_ coating more persistent under our acidic conditions than the Ni­(OH)_2_ coating more soluble due to the inability of Ni^2+^ ions to undergo further oxidation.^55,56^ Consequently, ZVI@MP@Ni are less effectively protected over time, exhibiting behavior similar to that of particles that are not coated with hydroxide after 24 h. This study provides strong evidence that the ZVI@MP@Mn sample is the most suitable candidate for applications in Fenton catalysis under mildly acidic conditions. From a corrosion chemistry perspective, the observed differences in stability between Ni­(OH)_2_- and Mn­(OH)_2_-coated ZVI nanocatalysts can be attributed to the redox behavior of the respective metal ions. Mn^2+^ is more prone to further oxidation, potentially forming insoluble oxides such as MnO_2_, which can act as a protective barrier and enhance passivation. In contrast, Ni^2+^ does not readily oxidize under the same conditions, making Ni­(OH)_2_ coatings more susceptible to dissolution in acidic media. These differences in corrosion resistance directly influence the long-term stability and ROS generation capacity of the coated nanocatalysts. In Figure 7b, the fluctuation in ^•^CH_3_ kinetics for ZVI@MP@Mn (increased at day 1, decreased at day 5, and then increased again at day 7) can be attributed to the interaction between the buffer and hydrogen peroxide or between DMPO and hydrogen peroxide, leading to the formation of ^•^CH_3_ radicals. It is important to note that these radicals are secondary products and not the primary focus of this study, which is centered on the generation of hydroxyl radicals (^•^OH). While manganese-containing nanocatalysts are known to contribute to hydroperoxyl radical (^•^OOH) formation in Fenton-like systems, our EPR measurements showed a dominant signal for (^•^OH), making it difficult to resolve the ^•^OOH contribution. Therefore, our analysis focused on Fe^2+^ species, which are the primary drivers of ^•^OH generation under the experimental conditions. Although Mn may enhance ROS generation indirectly, its specific contribution could not be quantified due to signal overlap.

We also investigated the aging behavior of ZVI nanocubes coated exclusively with PVP in different proportions (ZVI@P1 and ZVI@P2). Based on magnetic characterization data indicating significant nanoparticle degradation within 24 h of suspension in an acetate buffer, the kinetics of ^•^OH radical generation from these samples were limited to the initial suspension stage (0 h) and after 24 h in buffer. The findings are depicted in Figure 8, illustrating the important changes in ^•^OH radical production associated with the observed degradation over time.

**Figure 8:**
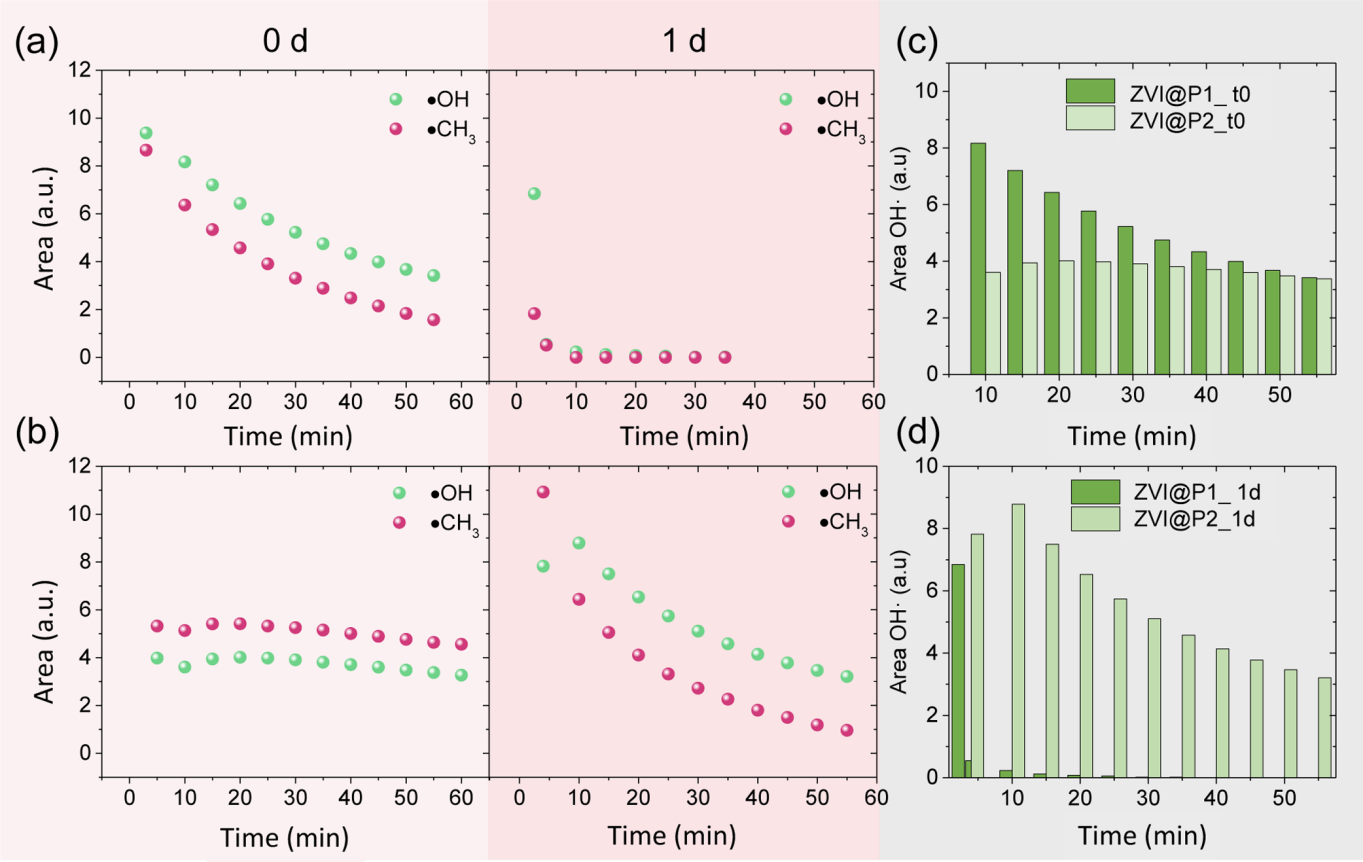
Kinetic curves of the free radicals produced by ZVI@P1 (a) and ZVI@P2 (b) measured at different aging times (0, 1 d) in buffer pH 5. Comparison of the kinetic studies of the free hydroxyl radicals catalyzed by ZVI@P1 and ZVI@P2 at different aging times in buffer pH 5: 0 d (c) and 1 d (d). All EPR measurements were normalized using a MgO/Mn^2+^ reference, and the estimated experimental uncertainty in ROS determination is within ±10%.

The metallic ZVI nanocubes coated with PVP polymer display different behaviors in radical generation. The ZVI@P1 sample follows a pattern analogous to ZVI@MP@Ni, characterized by an initial production of ^•^OH radical upon hydrogen peroxide addition, followed by a decline to approximately 60% after 60 min. In contrast, the ZVI@P2 sample demonstrates a consistent trend in radical generation. Specifically, after 60 min from the addition of hydrogen peroxide, the quantity of ^•^OH radicals in the medium remains nearly unchanged compared with the start of the kinetic study. The amount of ^•^OH radicals at 0 and 1 d for each sample has been quantified and compared in Figure 8c. This disparity in the catalytic behavior can be attributed to the morphology and structure of each sample. As a result of the characterization by SEM and TEM microscopy, the ZVI@P1 sample features metallic cubes with minimal coating, leaving the iron atoms exposed and vulnerable to oxidation by hydrogen peroxide in an acidic environment. Conversely, the ZVI@P2 sample encases these iron atoms within a protective polymeric envelope, shielding them from rapid oxidation. The removal of PVP combined with the acidic conditions, leads to rapid oxidation of exposed iron atoms, to Fe^3+^ as Fe_2_O_3_ in the ZVI@P1 sample, resulting in diminished ^•^OH radical production. In contrast, the ZVI@P2 sample’s polymer envelope preserves the integrity of the metallic cubes, preventing premature oxidation, ensuring sustained catalytic activity for longer times.

Uncoated ZVI nanoparticles were not included in the EPR/Fenton tests due to their extremely poor stability under the experimental conditions employed, specifically in acetate buffer at pH 5. Upon dispersion in this mildly acidic medium, the particles undergo immediate oxidation, which prevents the acquisition of meaningful kinetic data. This rapid degradation highlights the necessity of surface passivation strategies and supports the rationale for focusing this study on coated ZVI nanocatalysts capable of maintaining catalytic activity under such conditions.

After the nanocatalysts were subjected to a 24 h aging period in acetate buffer, their catalytic activity exhibited distinct behaviors. The ZVI@P1 sample showed a drastic reduction in ^•^OH radical production immediately upon addition of hydrogen peroxide, reaching near-zero production after 5 min due to complete oxidation of the iron to Fe^3+^. In contrast, the ZVI@P2 sample maintained catalytic activity even 24 h after dispersion in acidic media, pH 5. However, the production of ^•^OH radicals was no longer constant, exhibiting higher activity at the beginning compared with the unaged sample and a decline over time. This clearly points to a less protected surface after aging for 24 h, allowing for gradual oxidation of the nanocubes to occur during the test. This finding underscores the crucial role of the microstructure of the polymer coating in preserving the catalytic activity and delaying the oxidation of ZVI nanocatalysts under acidic conditions. While a thermodynamic evaluation could provide additional insight into the redox equilibrium and degradation feasibility of iron-based systems, in this work we focused on the experimental kinetics of ROS generation resulting from the Fenton-like interaction between Fe^0^ and H_2_O_2_. This approach allowed us to directly correlate the oxidation of Fe^0^ to Fe^2+^/Fe^3+^ with observed radical production and catalyst stability. Nevertheless, thermodynamic analyses have been extensively discussed in literature as a means to estimate the degradation capacity and reaction equilibrium of Fenton-like systems, and such approaches could complement our experimental findings in future studies.^57^

A concise overview of the sample codes, coating combinations, observed stability in acidic media, and ROS generation ability based on EPR measurements is provided in Table 2. Additional structural parameters, including the estimated degree of crystallinity (DOC %) and crystallite size derived from XRD analysis, are provided in the Supporting Information (Table S1). A higher DOC % generally enhances electron mobility and structural integrity, which are critical for sustaining catalytic activity in Fenton-like reactions. This explains why highly crystalline samples, such as ZVI@MP and ZVI@MP@Ni, maintain strong ROS generation over time. However, the same high reactivity that promotes efficient electron transfer also accelerates interaction with hydrogen peroxide, favoring surface oxidation. The formation of iron oxide layers, particularly in samples with prolonged exposure to acidic media (e.g., ZVI@P2), introduces amorphous regions and reduces the overall DOC %. This decrease in crystallinity can disrupt electron transfer pathways and limit the catalytic efficiency in subsequent cycles. Therefore, while high DOC % correlates with superior initial performance, it may also lead to faster structural changes due to oxidation, highlighting a trade-off between short-term activity and long-term stability.^58^

**Table 2:** Summary of ZVI Nanocatalyst Samples: Coating Composition, Stability, and ROS Generation

sample code	coating type	stability in acidic media (pH 5)	ROS generation (^•^OH via EPR)
ZVI@MP	mannitol + PVP	low (degraded after 1 day)	high at 0 h, drops rapidly
ZVI@MP@Ni	mannitol + PVP + Ni(OH)_2_	low (degraded after 1 day)	high at 0 h, drops after
ZVI@MP@Mn	mannitol + PVP + Mn(OH)_2_	high (stable up to 7 days)	moderate but sustained
ZVI@P1	PVP (low content)	low (degraded after 1 day)	moderate, drops after 1 day
ZVI@P2	PVP (high content)	moderate (stable after 1 day)	consistent over time

Due to the irreversible oxidation of ZVI nanocatalysts under the experimental conditions employed (acetate buffer, pH 5), recovery and reusability tests were not feasible. The rapid degradation of the iron core, particularly in samples with insufficient coating, prevents the regeneration of catalytic activity after the reaction. Therefore, this study focused on evaluating the initial stability and ROS production capacity of coated ZVI nanocatalysts. Future work will explore reusability under milder conditions where recovery may be possible.^59^

Continuous or higher-frequency monitoring would provide a more accurate understanding of the degradation kinetics as transient oxidation events can significantly affect the catalytic performance.^60^ Moreover, leaching of metal ions from the coatings or the ZVI core itself may contribute to both activity loss and environmental impact. Investigating leaching behavior is essential to assess long-term stability and safety, as highlighted in recent studies on nanoparticle-based remediation systems.^61^

## 4 Conclusions

In conclusion, this study evaluated the stability and catalytic performance of zerovalent iron (ZVI) nanocatalysts in AOPs for environmental remediation (e.g., wastewater treatment), emphasizing the role of polymer and metal hydroxide passivation strategies. Our findings indicate that the doubly coated ZVI nanocatalysts with mannitol–PVP and nickel hydroxide, and simply coated ZVI nanocatalysts with PVP and mannitol–PVP undergo oxidation upon storage in acetate buffer, resulting in a significant decline in catalytic activity after 1 d. Despite the observed oxidation, these particles exhibit higher catalytic activity to produce reactive oxygen species compared with other materials like iron oxide nanocatalysts, which would significantly reduce the amount of catalyst required in an application. This enhanced activity was also supported by EPR measurements, which showed a dominant signal for hydroxyl radicals (^•^OH), confirming the role of Fe^2+^ as the main ROS generator under the tested conditions. On the contrary, doubly coated ZVI nanocatalysts with mannitol–PVP and manganese hydroxide, and simply coated ZVI nanocatalysts with high PVP quantity retained catalytic activity even after being suspended in water at pH 5 for 24 h, the Mn-bearing particles remaining stable for up to 7 days. TEM analysis revealed that these nanocatalysts preserved their morphology after the reaction, supporting their structural stability. Notably, the highly PVP-coated ZVI sample exhibited consistent catalytic activity over time, suggesting that PVP is capable of effective protection of iron atoms from oxidation without affecting the catalytic properties of ZVI. Overall, this study demonstrates the successful development of stable zerovalent iron nanocatalysts capable of withstanding oxidation processes in acidic environments, thereby enhancing their potential for practical applications in Fenton-like water purification systems and providing design guidelines for durable nanocatalysts based on polymers and metal hydroxide passivation strategies.

## Supplementary Material



## Notes

Structural and magnetic characterization of ZVI-based nanocatalysts (ZVI@MP, ZVI@MP@Mn, ZVI@MP@Ni, ZVI@P1, and ZVI@P2); TEM and HAADF-STEM images revealing morphology and spinel-type oxide shells; EELS line-scan confirming ∼3 nm Fe oxide surface layer; size distribution histograms and summary table of TEM size, crystal size, and degree of crystallinity; thermogravimetric profiles of polymer-passivated nanoparticles (ZVI@P1 and ZVI@P2); magnetic characterization including hysteresis loops at RT and 5 K for Mn- and Ni-modified systems; and coercivity and *M*_R_/*M*_S_ ratios (PDF)

## Abbreviations

AOP: advanced oxidation process
EPR: electron paramagnetic resonance
ROS: reactive oxygen species
PVP: polyvinylpyrrolidone
ZVI: zero-valent iron.
