# Supplementary material for: Zero-Valent Iron Nanocatalysts via Polymers or Metal Hydroxide Passivation: Implications for Advanced Oxidation Processes
Source: ACS Appl Nano Mater. 2025 Nov 29;8(49):23527–40. doi: 10.1021/acsanm.5c04286 (PMC12706743; doi:10.1021/acsanm.5c04286)
Supplement: Supplementary file 1 [file an5c04286_si_001.pdf]

Supporting information

Zero-Valent Iron Nanocatalysts via Polymer or Metal Hydroxide Passivation: Implications for Advanced Oxidation Processes

Carlos Díaz-Ufano [a,b*], *Nahuel Nuñez* [b,c,d,e], *Alvaro Gallo-Cordova* [a], *Elin L. Winkler* [c,d,e*], *María del Puerto Morales* [a], *Sabino Veintemillas-Verdaguer* [a*]

*Corresponding Autor Email: carlos.diazufano@urjc.es; elin.winkler@ib.edu.ar; sabino@icmm.csic.es.

[a] Departamento de Nanociencia y Nanotecnología, Instituto de Ciencia de Materiales de Madrid, ICMM/CSIC, C/Sor Juana Inés de la Cruz 3, 28049 Madrid, Spain.

[b] Escuela de Doctorado UAM, Centro de Estudios de Posgrado, Universidad Autónoma de Madrid. C/ Francisco Tomás y Valiente, 2, 28049, Madrid, Spain.

[c] Laboratorio de Resonancias Magnéticas, Gerencia de Física, Centro Atómico Bariloche, Av. Bustillo 9500, (8400) S. C. de Bariloche (RN), Argentina.

[d] Instituto de Nanociencia y Nanotecnología, CNEA/CONICET, Nodo Bariloche, Av. Bustillo 9500, (8400) S. C. de Bariloche (RN), Argentina.

[e] Instituto Balseiro, CNEA-UNCuyo, Av. Bustillo 9500, (8400) S. C. de Bariloche (RN), Argentina.

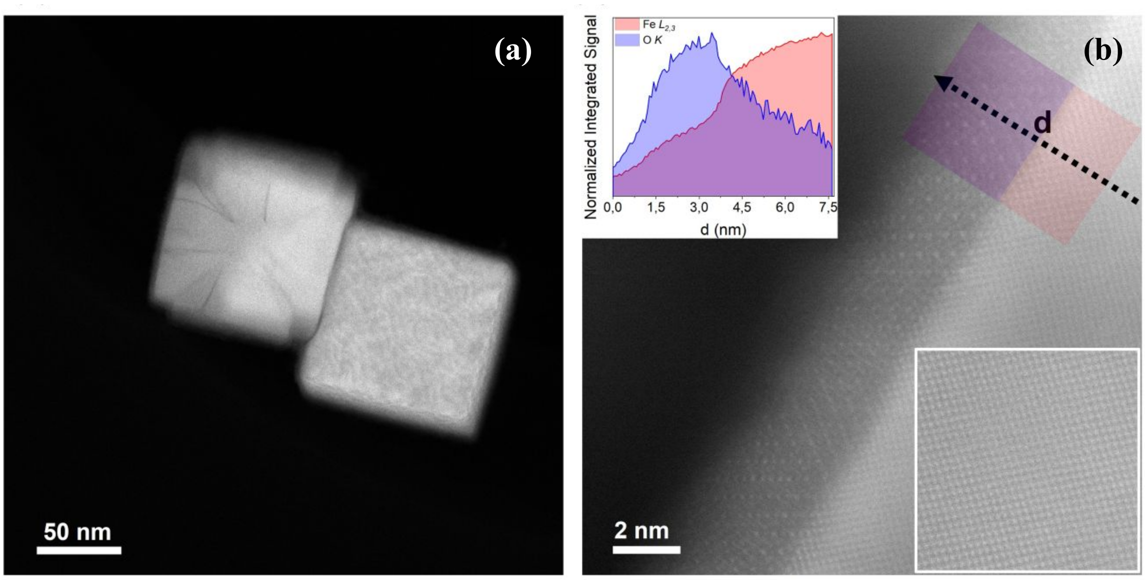

Figure S1: HAADF-STEM images of the nanoparticles. (a) Low-magnification view of the squared-shaped Fe nanoparticles. (b) Atomic resolution image of the nanoparticle edge revealing a spinel oxide superficial layer. The lower right inset focuses on the nanoparticle core, while the upper left inset presents the normalized integrated EELS signal of Fe $L_{2,3}$ (red) and O K (blue) edges along a linescan (dashed arrow), confirming the formation of a Fe oxide layer of approximately 3 nm.

Table S1: TEM sizes, Crystal sizes and degree of crystallinity of the samples synthetized

Sample	TEM size (nm)	Crystal size (nm)	DOC (%)
ZVI@MP	80 ± 20	32 ± 2	95-98
ZVI@MP@Mn	80 ± 20	32 ± 2	90-95
ZVI@MP@Ni	80 ± 20	32 ± 2	95-98
ZVI@P1	153 ± 45	50 ± 3	85-90
ZVI@P2	249 ± 85	70 ± 4	80-85

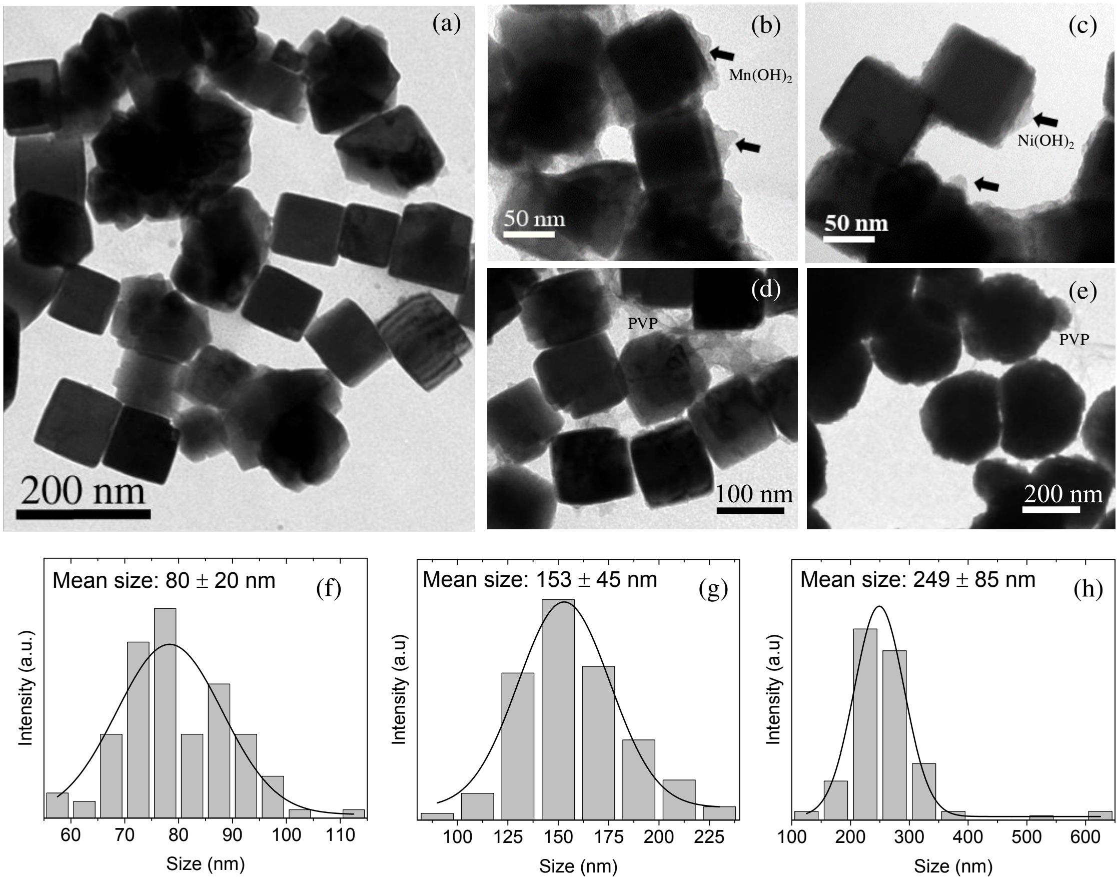

Figure S2: TEM micrographs of ZVI@MP (a), ZVI@MP@Mn (b), ZVI@MP@Ni (c), ZVI@P1 (d), and ZVI@P2 (e). Size distribution of the samples MP@ZVI (f), P1@ZVI (g) and P2@ZVI (h)

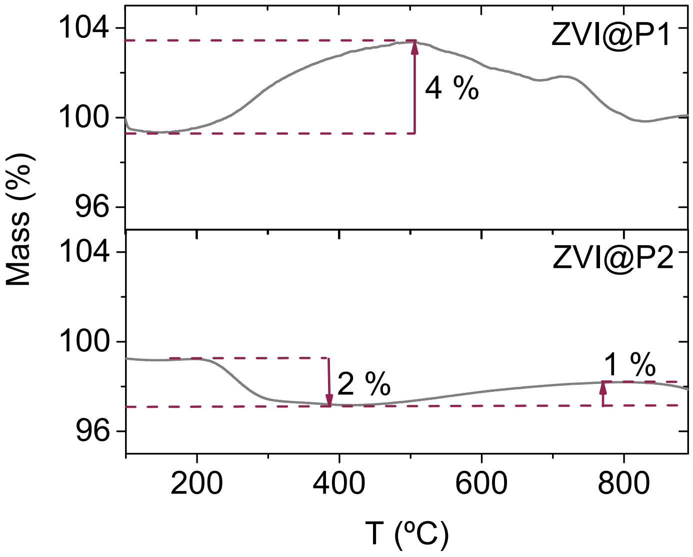

Figure S3: Thermogravimetrical analysis of the samples ZVI@P1 (up) and ZVI@P2 (down)

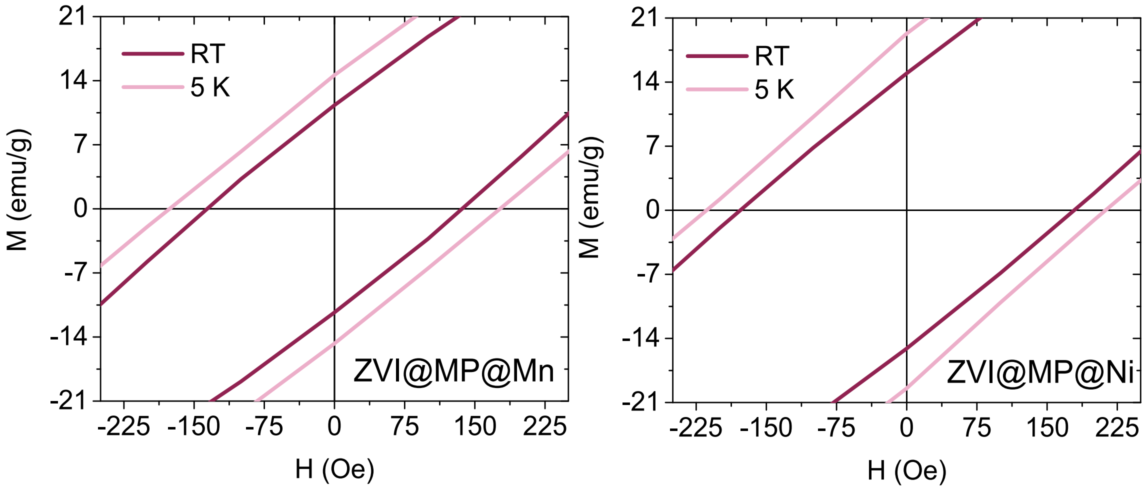

Figure S4: Hysteresis loops of the samples ZVI@MP@Mn (left) and ZVI@MP@Ni (right) at room temperature (RT) and 5 K.

Table S2: Magnetic measurements at 5 K and RT.

Sample	M_R/M_S (5 K)	H_c (RT) / Oe	H_c (5K) / Oe
ZVI@MP@Mn	0.08	140	175
ZVI@MP@Ni	0.08	185	215
